# A Prospective, Longitudinal Study of Caregiver-Reported Adaptive Skills and Function of Individuals with *HNRNPH2*-related Neurodevelopmental Disorder

**DOI:** 10.1007/s41252-023-00346-1

**Published:** 2023-08-07

**Authors:** Thomas J. Davis, Rachel Salazar, Sarah Beenders, Amelia Boehme, Nicole M. LaMarca, Jennifer M. Bain

**Affiliations:** 1 Department of Neurology, Columbia University Irving Medical Center and NewYork Presbyterian Morgan Stanley Children’s Hospital, New York, NY USA; 2 Department of Interprofessional Health Sciences and Health Administration, Seton Hall University, Nutley, NJ United States; 3 Department of Physical Therapy and Rehabilitation Sciences, Drexel University, PA Philadelphia, United States

**Keywords:** HNRNPH2, Neurodevelopmental Disorders (NDD), Adaptive Function, Genetics, Neurology

## Abstract

**Objectives:**

This study presents a cohort of individuals in a natural history study with de novo pathogenic missense variants in *HNRNPH2* causative of *HNRNPH2*-related neurodevelopmental disorder (NDD) to describe individuals’ adaptive functional abilities.

**Methods:**

We measured adaptive function using the Pediatric Evaluation of Disability Inventory Computer Adaptive Test (PEDI-CAT) and the Vineland Adaptive Behavior Scale (VABS-III). Results were compared using inferential statistics and regression analysis.

**Results:**

Sixty-seven individuals carried known pathogenic or likely pathogenic variants in *HNRNPH2*. Thirty-five participants (2.89–42.04 years, 83% female) and caregivers completed PEDI-CAT assessments with 25 of these participants completing the VABS-III. Sixteen, three and two participants completed a follow-up PEDI-CAT assessment at one, two and three years respectively. Individuals had mean normative scores less than age-matched peers across all domains on both PEDI-CAT and VABS-III measures, with 91% participants < 5^th^ percentile on both the PEDI- CAT and VABS-III. Verbal and ambulatory participants had significantly higher PEDI-CAT scores across all domains, using both raw and normative data. There was no significant change in PEDI-CAT scores over 3 years.

**Conclusions:**

Overall scores, both raw and normative, are low across all individuals with *HNRNPH2*-related NDD using both the PEDI-CAT and VABS-III. PEDI-CAT normative scores do not likely represent the clinical variability, but raw scores may be able to capture functional variability. In a small sample, longitudinal data from the PEDI-CAT domain scores demonstrate stability in performance at 3 years.

**Trial Registration**: ClinicalTrials.gov NCT03492060.

As sequencing technology improves and the cost of genome sequencing decreases, rare causal genetic variants are identified in 10–30% of individuals with neurodevelopmental disorders (NDDs) such as autism spectrum disorder (ASD) or intellectual disability (ID) (Hu et al., 2014; Manickam et al., 2021; Vorstman et al., 2017). As NDDs encompass a variety of motor and cognitive deficits, they can carry with them a profound burden for both individuals and caregivers with limited therapeutic interventions identified for most of the rare and ultra-rare genetically identified NDDs. It is essential to deeply characterize the phenotypic spectrum, describe the natural history of the disorder and develop meaningful clinical outcome measures for clinical trial readiness (Hu et al., 2014; Krishnan et al., 2021). Indeed the FDA has identified that particularly in rare diseases, longitudinal natural history studies are a way to identify subgroups and prognostic factors, as well as identify clinical outcome assessments (*Rare Diseases: Natural History Studies for Drug Development*, 2019). Furthermore, caregivers for individuals with NDDs have shown interest in participating in clinical research registries and providing patient reported feedback about clinically meaningful outcome measures (Bain et al., 2021a, b, 2022; Kalb et al., 2019).

In 2016, pathogenic variants were identified in *HNRNPH2* (GenBank: NM_019597.4) located at Xq22.1, in association with a NDD characterized by developmental delay/intellectual disability, autistic features, hypotonia, motor and gait disturbances, and seizures (*HNRNPH2*-related NDD or Intellectual Developmental Disorder, X-linked syndromic, Bain type, OMIM 300986) (Bain et al., 2016, 2021a, b). The initial case series identified 6 females with a cluster of predicted pathogenic missense variants within the nuclear localization sequence (NLS) of the encoded heterogenous nuclear riboprotein H2 (*HNRNPH2)* (Bain et al., 2016). Since the initial description, the cohort has expanded in number of individuals being identified. Additionally, other pathogenic variants have been identified, including the identification of variants outside of the NLS and variants being diagnosed in several males, despite an initial hypothesis that variants were embryonic lethal in males (Bain et al., 2021a, b; Gillentine et al., 2021; Harmsen et al., 2019; Jepsen et al., 2019; Kreienkamp et al., 2022). As more individuals are identified, the clinical spectrum of the phenotype is widening, and a deeper characterization becomes essential in understanding the landscape and trajectory of this disorder.

Two difficulties in studying NDDs and treatment responses in intervention trials, are (1) difficulty identifying clinically meaningful outcome measures and (2) monitoring for meaningful changes in function. Adaptive function is studied as often the measurement of intellectual functioning alone is insufficient to appropriately characterize the spectrum of the disorder. For these individuals, clinical improvement may be more effectively measured by describing their adaptive skills. Adaptive skills or behaviors are defined as conceptual, social, and practical skills that are performed by people as activities of daily living (ADLs) (Tasse et al., 2012). Many performance-based outcomes need to be developed to provide quantitative measures as an assessment of ADLs. However, due to the clinical variability and comorbidities in individuals with NDDs, performance-based evaluations need to be evaluated to see if they are appropriate for use in specific populations (Krishnan et al., 2021). Furthermore, parent-reported measures can provide a proxy to collect validated data representative of adaptive and cognitive skillsets, in addition to highlighting potential challenges. Previous work has focused on identifying salient motor impairments in the H2 population described using the gait analysis in combination with measures of adaptive function (Duong et al., 2020; Salazar et al., 2021), however there is a need to more deeply characterize the adaptive function of *HNRNPH2*-related NDD using parent-reported measures recognizing the significant motor challenges in the group.

For the pediatric population, the Vineland Adaptive Behavior Scale III (VABS-III) and the Pediatric Evaluation of Disability Inventory- Computer Adaptive Test (PEDI-CAT) are two commonly used parent-report measures for assessment of the functional status of individuals looking at their strengths and limitations in adaptive behavior. The VABS-III measures three domain level scores, Communication, Daily Living Skills, and Socialization in addition to a composite score, the Adaptive Behavior Composite (ABC). The Daily Living Skills domain assesses the subject's performance of the practical, everyday tasks of living that are appropriate for his/her age, containing 3 subdomains: personal, domestic and community. The Socialization domain reflects the functioning in social situations with 3 subdomains: interpersonal relationships, play and leisure and coping skills. In addition to these domains, there is a Motor Skills domain measuring Gross motor and Fine motor, but this domain is only normed through the age of 9 years. Each domain, and the composite ABC are given raw scores and norm-based standard scores with a mean of 100 and standard deviation of 15. The VABS-III is a commonly used measure of adaptive behavior that has been extensively studied and has been used to understand ASD, Down syndrome and many other NDDs (Farmer et al., 2020; Garrido et al., 2017; Hamburg et al., 2019; Mouga et al., 2015). Importantly, however, it is appreciated that many individuals with NDDs have low norm-based scores that would demonstrate a basement effect instead of showing a more diverse stratification within the group. In addition, the VABS-III norm-based scores may poorly represent the longitudinal course of skill acquisition in NDDs and using raw scores or growth score values may be more indicative of growth in lieu of deterioration noted by scaled or normative sampling (Berg et al., 2021; Levine et al., 2022).

The PEDI-CAT assessment has previously been validated for evaluating adaptive function in children with ASD, cerebral palsy, and others who are medically complex (Coster et al., 2016; Dumas et al., 2017; Shore et al., 2019). Salazar and Bain have previously shown convergent validity between the parent-reported PEDI-CAT and clinician-administered Gross Motor Function Measure (GMFM-88) in *HNRNPH2*-related NDD (Salazar et. al, 2021). The PEDI-CAT measures four functional domains: Daily Activities, Motor Ability, Cognitive/Social, as well as a Responsibility domain, which measures the extent a child is managing tasks of living independently. For each of the four domains, individuals are given scaled scores which are not based on age and can be measured over time as well as normative standard scores (provided as T-scores with age percentiles). For T-scores, the mean for each age group is 50, with a standard deviation of 10, with T-scores between 30 and 70 (i.e. mean ± 2 standard deviations) are considered within the expected range for age. Scores below 30 indicate decreased functional ability compared to what is typically expected for that age range. Scores above 70 indicate scores above what is typically expected for that age range. Importantly, the PEDI-CAT can be used through 21 years of age and can change over time in children, unlike a measure such as intelligence quotient (Dumas et al., 2012). The PEDI-CAT is not a capacity-based assessment but rather a measure of overall functional activity performance and items are written to focus on the outcome of activity performance, allowing a variety of methods to be used given their abilities and challenges. Notably, items do not require the child to perform the activity in a standardized manner so individuals can complete activities using alternative methods and won’t “penalize” individuals who use of adaptations or technology to function (e.g. augmentative communication devices, switches). In addition, the PEDI-CAT has been used to show longitudinal changes in groups of individuals with cerebral palsy across various functional domains (Burgess et al., 2020; Burgess et al., 2022a, b). Salazar et al. (2021) showed a strong correlation between caregiver-reported PEDI-CAT Mobility and clinician-observed motor skills using the Gross Motor Function Measure-88 in *HNRNPH2*-related NDD.

The goal of this study is to both present the expanded cohort with the identified genetic variants using both the VABS-III and PEDI-CAT to understand each of their applicability in the cohort of *HNRNPH2*-related NDD. Prior work showed all norm-based domain and composite VABS-III scores were below the 5 percentiles for 33 individuals (Bain et al., 2021a, b). For this study, we included several older individuals, and sought to capture the motor function that would be above the age range for VAB-III norm-based motor measures and instead used the PEDI-CAT to assess the motor domain in individuals up to 21 years old. We further sought to identify whether there are specific subgroups that may have better adaptive functioning or longitudinal outcomes.

## Methods

### Participants

Participants are enrolled in a prospective observational natural history study if they were diagnosed with pathogenic or likely pathogenic variants in *HNRNPH2*. These variants were identified using American College of Medical Genetics and Genomics/Association of Molecular Pathology Criteria, mainly using clinical exome sequencing. Thirty-five participants completed PEDI CAT evaluations. This population consisted of 29 female and 6 male participants. The mean age of participants were 14.32 years old with range of participant age were from 2.89- 42.04 years of age.

### Procedure

Caregivers for all enrolled individuals completed VABS-III edition and PEDI-CAT tool using an online platform. Subjects and their caregivers were asked to complete both surveys upon enrollment and then annually.

Recognizing that the term nonverbal can be categorized differently in the research domain, we chose to use the recommendations from Koegel et al. and the term nonverbal in this study was based upon various sources of language including natural interactive communication, sampled optimally with a familiar communication partner in addition to caregiver reports (Koegel et al., 2020; Posar & Visconti, 2021). We considered nonverbal as individuals over 18 months of age demonstrating no consistent verbal expressive words (intelligible or approximations) during standardized tests, across settings during observations, and according to parent report. In addition, we considered minimally verbal individuals who used some words, but significantly fewer than expected levels relative to age. We did not differentiate between nonverbal and minimally verbal, as the intention was not to present a formal language and communication evaluation for this study, and combined the likely distinct sub-groups.

### Measures

The standardized measures that we used were the Vineland Adaptive Behavior Scale – 3^rd^ edition (VABS-III) and the Pediatric Evaluation of Disability Inventory- Computer Adaptive Test (PEDI-CAT). For the VABS-III, individuals are given raw scores and then raw scores are converted into standard scores for five major domain composite scores: Communication, Daily Living Skills, Socialization, Adaptive Behavior Composite, and Motor (for individuals under nine years old). For these domains, a standard score (T-score) of 100 is the mean with a standard deviation of 15 points.

The PEDI-CAT provides measures in four functional domains: Daily Activities, Motor Ability, Cognitive/Social, as well as a Responsibility domain, which measures the extent a child is managing tasks of living independently. For each domain, an individual is given two scores: a normative score based upon age-appropriate scores (presented as a T-scores) and a scaled score which does not reflect age-based scores. For normative T-scores, the mean for each age group is 50, with a standard deviation of 10, with T-scores between 30 and 70 (i.e. mean ± 2 standard deviations) considered within the expected range for age. The PEDI-CAT’s normative standardization sample was recruited through an online panel with a representative sample of 2,205 parents of children less than 21 years of age in the contiguous United States. Scaled scores provide a way to look at a child’s current functional skills and progress in these skills over time. In contrast, the scaled scores are not age-based and can be used to document improvements in functional skills for children not expected to exhibit or regain normative levels of functioning.

### Data Analyses

T-tests were used to compare between groups with significance denoting p < 0.05 or otherwise noted. Descriptive statistics for the VABS-III and PEDI-CAT were performed on participants scaled scores at baseline and at one-year follow up intervals. Follow up mean domain score differences were analyzed using paired T-tests. We calculated correlation coefficients between the Vineland Composite score and the PEDI-CAT Scaled Domain within the functional domains and assessed for differences between individual populations based on phenotypic (verbal versus non-verbal and ambulatory versus non-ambulatory participants) and genotypic (inside and outside the NLS) variation.

## Results

**Fig. 1:**
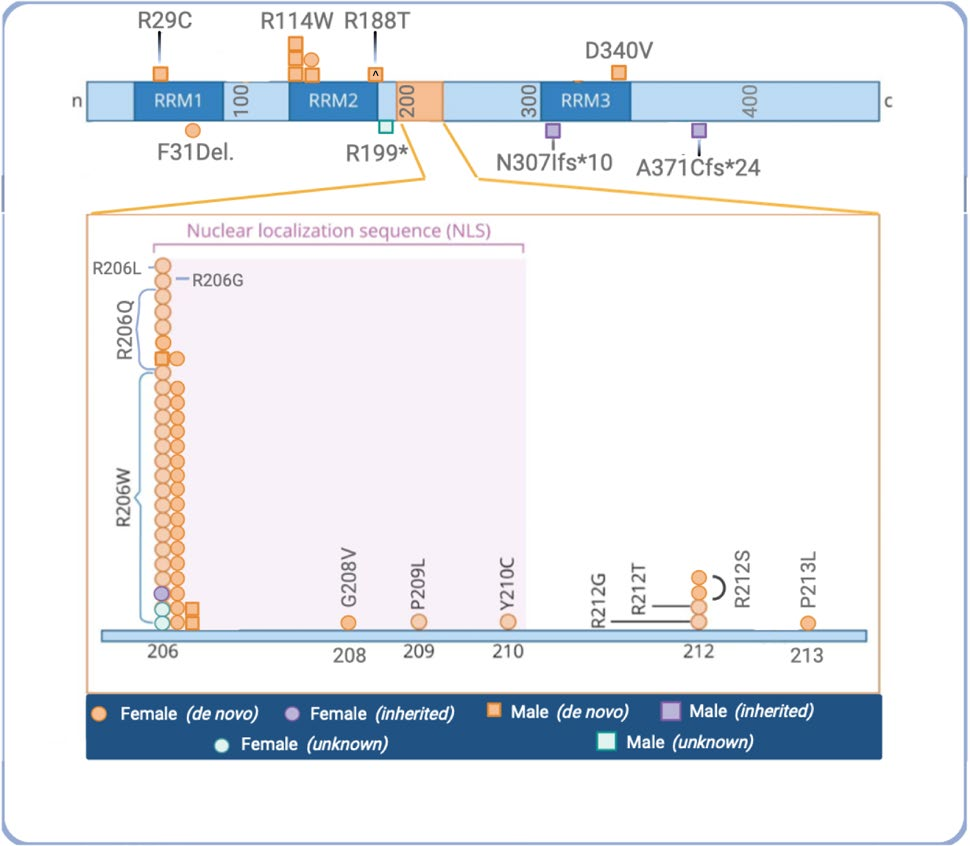
Full genotype map of the participants with known pathogenic variants in HNRNPH2. Each icon represents one individual. The Nuclear Localization Sequence (NLS) region is expanded given the density of pathogenic variants in this region. ^ denotes twin boys with same genotype R188T.

We identified sixty-seven individuals with pathogenic or likely pathogenic variants in *HNRNPH2* in the Natural History Study (Fig. 1).

### VABS-III Results

**Fig. 2:**
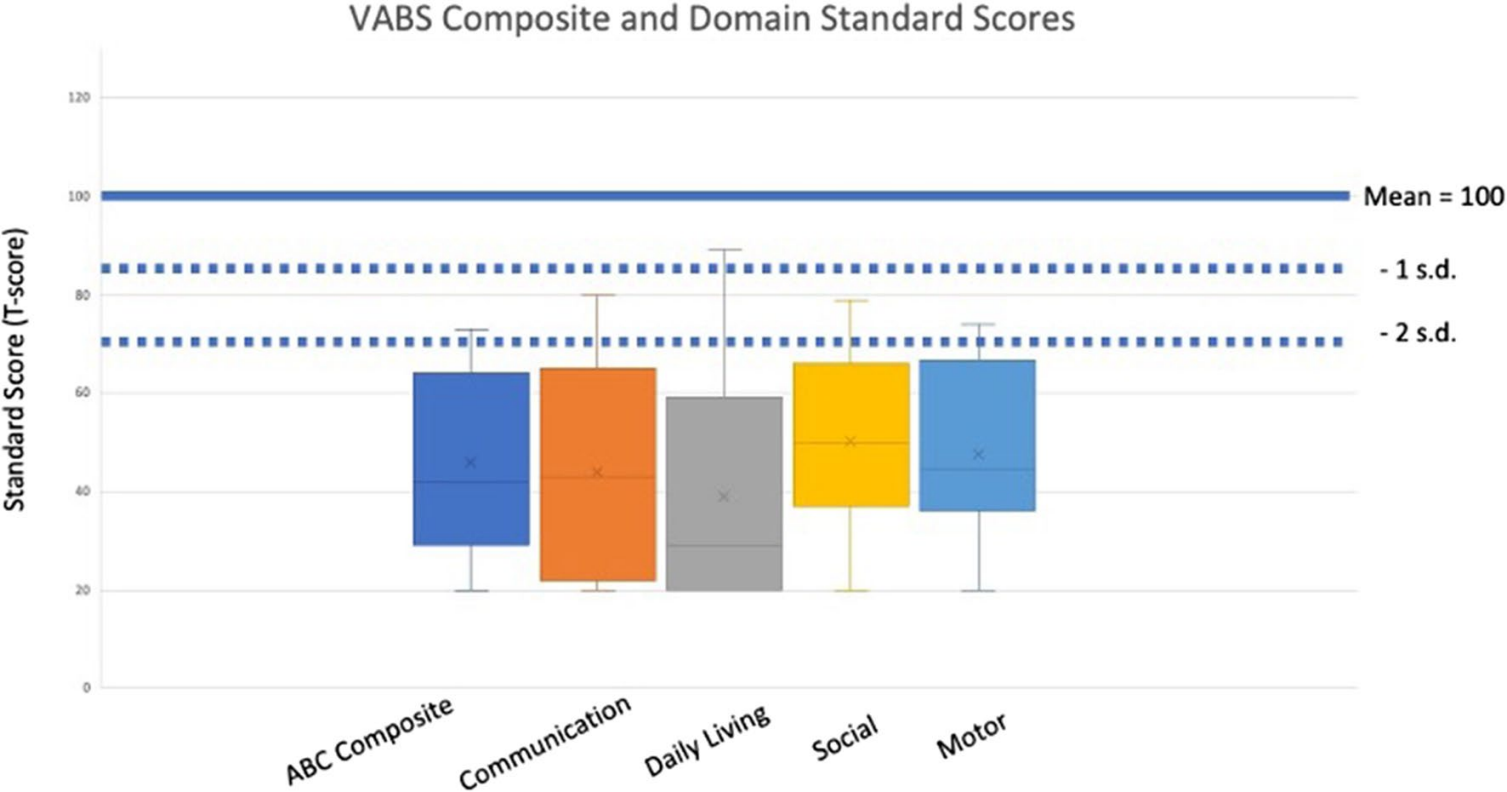
Vineland Adaptive Behavior Scale (VABS-III) mean scores. The left most bar represents the composite score (ABC), with the next four representing the individual domains. For each domain, error bars represent the 95% confidence interval, the bottom and top of the box are the 25th and 75th percentiles, the line inside the box is the 50th percentile (median). The dark blue line represents a normative mean of 100 with the 2 dotted lines representing 1 and 2 standard deviations below the mean. No significant difference was seen between these domains

Twenty-seven participants who had completed the PEDI-CAT, also completed the Vineland Adaptive Behavior Scales with an average age at the time of the completion of the survey of 15.52 years (Range of 1.76–42, Median of 11.34). VABS-III mean domain scores at baseline were Communication: 43.93 (SD 21.26), Daily Living Skills: 38.89 (SD 21.0), Socialization: 50.33 (SD 18.58), and ABC: 45.85 (SD 18.51) (Fig. 2.)

### PEDI-CAT Results

Thirty-five individuals completed PEDI-CAT assessments. The baseline results were analysed from 35 participants (Table 1) and consisted of 29 female and six male participants. The range of participant ages are from 2.89- 42.04 years old with a mean age of 14.32 years and a median age of 11.34 years. Of the 35 participants, 28.6% (*n* = 10) were verbal and 54.3% (*n* = 19) were ambulatory. One genotype was unknown at the time of analysis, leaving 34 individuals with known genotypes. In this cohort, 85.3% (*n* = 29/34) of these individuals had predicted pathogenic variants that fell within the previously identified NLS.

**Table 1:** Demographic information from Baseline PEDI-CAT Assessment

Demographics *N* = 35	
Age, range, years	2.89–42.04
Age, mean (SD), years	14.32 (10.92)
Median age, years	11.34
Gender, % (*n*), Female, Male	83% (29), 17% (6)
Total Genotype data *n* =	
Genotype, % (*n*), within NLS	77% (27)
- R206W	57% (20)
- R206Q	9% (3)
- R206L; R206G; P209L; Y210C;	12% (1 each)
Genotype, % (*n*), external to the NLS	20% (7)
- R188W	6% (2)
- R114W; R212T; P213L; D340V; A371CfsX24	15% (1 each)
Genotype, % (*n*), Unknown	3% (1)
Ambulatory Status, % (*n*), Ambulatory, non-ambulatory	54% (19), 46% (16)
Verbal Status, % (*n*), Verbal, non-verbal	29% (10), 71% (25)
Clinical Phenotype, % (*n*)	
Ambulatory and verbal	29%, (10)
Ambulatory and nonverbal	20%, (7)
Non-ambulatory and verbal	0%, (0)
Non-ambulatory and verbal	46%, (16)

Individuals with *HNRNPH2*-related NDD had mean normative scores less than age-matched peers across all domains. 91% of participants were below the 5^th^ percentile on the PEDI-CAT with 31 out of 36 participants falling below the 5^th^ percentile in all domains.

**Fig. 3:**
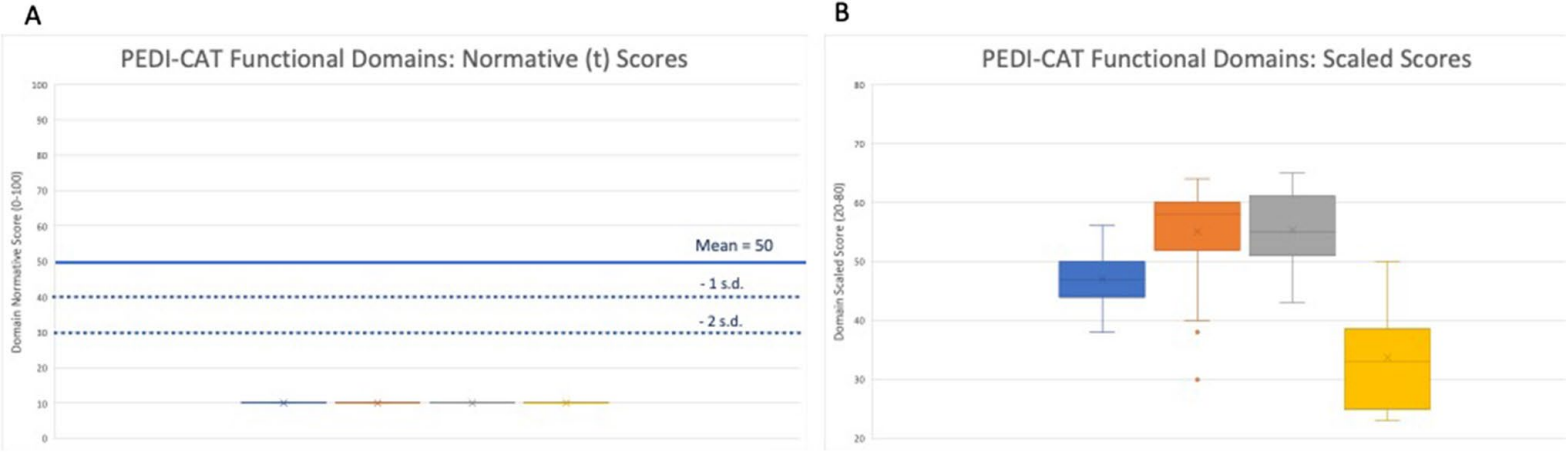
PEDI- CAT Mean Domain Normative (**a**) and Scaled (**b**) Scores. Each bar represents one of the four functional domains assessed by the PEDI-CAT survey. For each domain, error bars represent the 95% confidence interval, the bottom and top of the box are the 25th and 75^th^ percentiles, the line inside the box is the 50th percentile (median), and any outliers are shown as open circles. (A) For Normative scores, the dark blue line represents a normative mean of 100 with the 2 dotted lines representing 1 and 2 standard deviations below the mean

Next, we then looked at the scaled scores for PEDI-CAT functional domains. These scores are independent of norms based on age and represent performance at that time. PEDI-CAT mean domain scaled scores at baseline included Daily Activities: 47.03 (SD 4.44), Mobility: 55.06 (SD 7.72), social/cognitive: 55.26 (SD 5.69) and responsibility: 33.71 (SD 7.69). The Daily Activities domain scaled score was significantly lower than either the Mobility or the Social/Cognitive domain. The Responsibility scaled domain score significantly lower than the other three domains, tested using a paired two tailed T-Test (Fig. 3).

### VABS-III and PEDI-CAT Convergent Validity

We looked at the convergent validity between the domain scores of the PEDI-CAT and the VABS-III using Pearson correlation coefficient and paired two tailed T-test. Convergent Validity was shown between VABS-III Communication and PEDI-CAT Mobility (R = 0.33, *p* = 0.0042) and Social/Cognitive (R = 0.50, *p* = 0.0028); VABS-III Daily Living Skills and PEDI-CAT Daily activities (R = 0.41, *p* = 0.033), Mobility (R = 0.28, *p* = 1.6 × 10^–4^), and Social/Cognitive (R = 0.41, *p* = 1.0 × 10^–4^); VABS-III Socialization and PEDI-CAT Responsibility (R = 0.42, p = *p* = 6.1 × 10^–4^); and overall VABS-III ABC and Mobility (R = 0.19, *p* = 0.0080), Social/Cognitive (R = 0.37, *p* = 0.0052), and Responsibility (R = 0.51, *p* = 0.012) (Table 2).

**Table 2:** Convergent validity between PEDI-CAT scaled scores and VABS-III subcategories. PEDI-CAT Domains are denoted at the top with VABS-III categories and composite score (ABC) along the left. The boxes represent the R, the correlation coefficient with positive values representing positive correlation and negative values representing negative correlation. The closer to 1, the more strongly correlated these values were, and p was determined for significance. The comparisons in bold were significantly correlated, with *p* < 0.05

*N* = 27	**PEDI-CAT**: Daily activities	Mobility	Social/cognitive	Responsibility
VABS-III Communication	R = 0.44 *p* = 0.37	**R = 0.33** ***p* = 0.0042**	**R = 0.50** ***p* = 0.0028**	**R = 0.59** ***p* = 0.069**
Daily Living Skills	**R = 0.41** ***p* = 0.033**	**R = 0.28** ***p* = 1.6 × 10**^**–4**^	**R = 0.41** ***p* = 1.0 × 10**^**–4**^	R = 0.52 *p* = 0.68
Socialization	R = 0.17 *p* = 0.41	R = 0.097 *p* = 0.13	R = 0.22 *p* = 0.12	**R = 0.42** ***p* = 6.1 × 10**^**–4**^
ABC	R = 0.33 *p* = 0.66	**R = 0.19** p** = 0.0080**	**R = 0.37** ***p* = 0.0052**	**R = 0.51** ***p* = 0.012**
*N* = 10	**PEDI-CAT**: Daily activities	Mobility	Social/cognitive	Responsibility
VABS-III Mobility	**R = 0.74** ***p* = 0.014**	R = 0.58 *p* = 0.080	**R = 0.68** ***p* = 0.032**	**R = 0.70** ***p* = 0.025**

### Prognostic Factor Analysis of Baseline

**Fig. 4:**
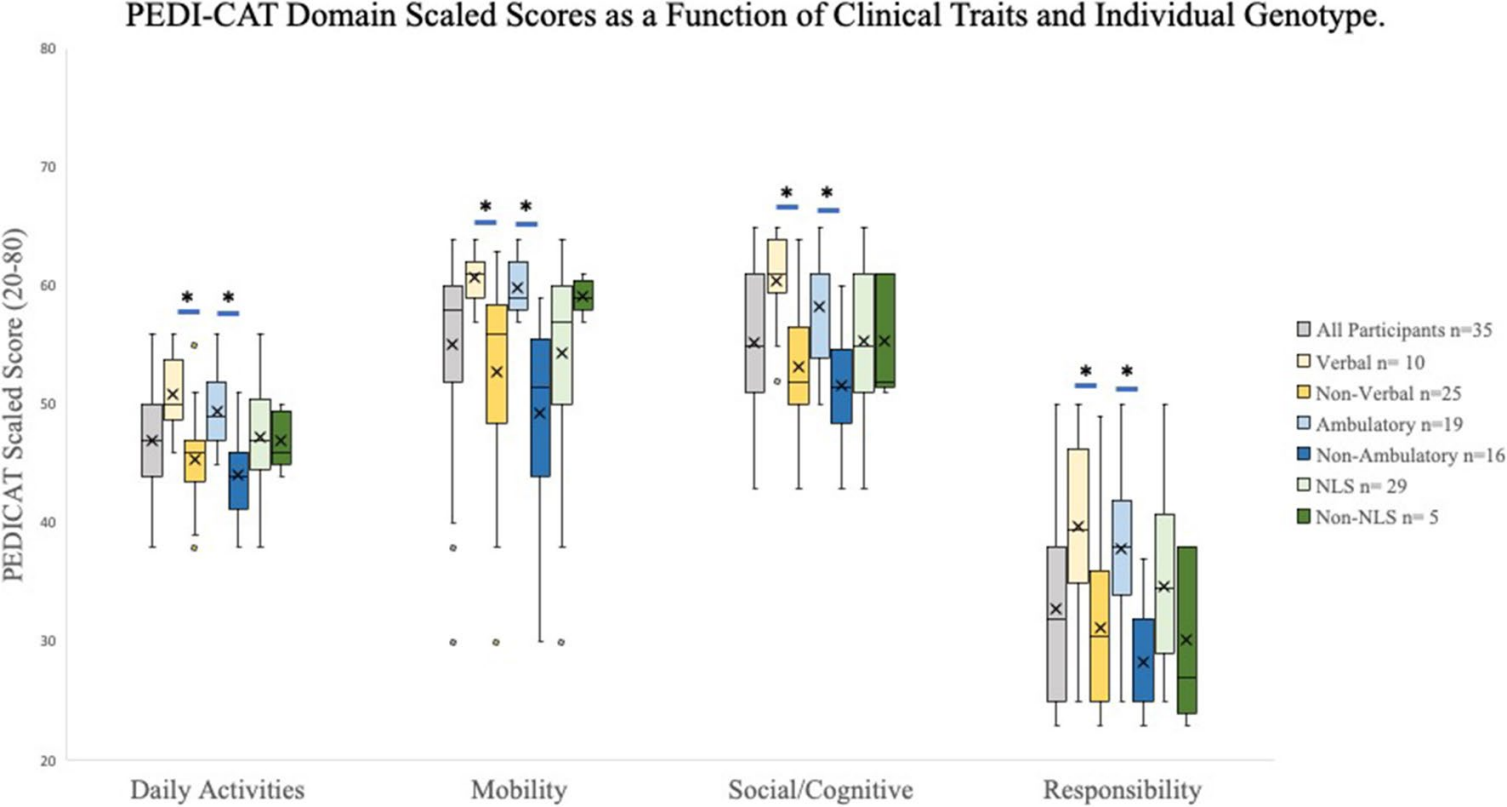
PEDI-CAT Domain Scaled Scores as a Function of Clinical Traits and Individual Genotype. For each domain, error bars represent the 95% confidence interval, the bottom and top of the box are the 25th and 75th percentiles, the line inside the box is the 50th percentile (median), and any outliers are shown as open circles. Horizontal bars represent significant difference as determined by two tailed T-Test. A significant difference was seen between verbal and ambulatory participants compared to their non-verbal and non-ambulatory peers across all domains. A significant difference was seen when comparing between genotype variant location (internal vs. external to the NLS) only in the mobility domain

When assessing scores based on clinical phenotype, ambulatory participants had statistically significantly higher PEDI-CAT domain scaled scores when compared to their non-ambulatory peers. There was also a significant difference between verbal and non-verbal patients in all four PEDI-CAT domains. In the comparison of participants with variants inside the identified NLS versus those external to it, there is no significant difference in Daily Activities, Social/Cognitive or Responsibility Domains. There was, however, a significant difference between those within and outside the NLS in the motor domain of the PEDI-CAT (Fig. 4).

**Fig. 5:**
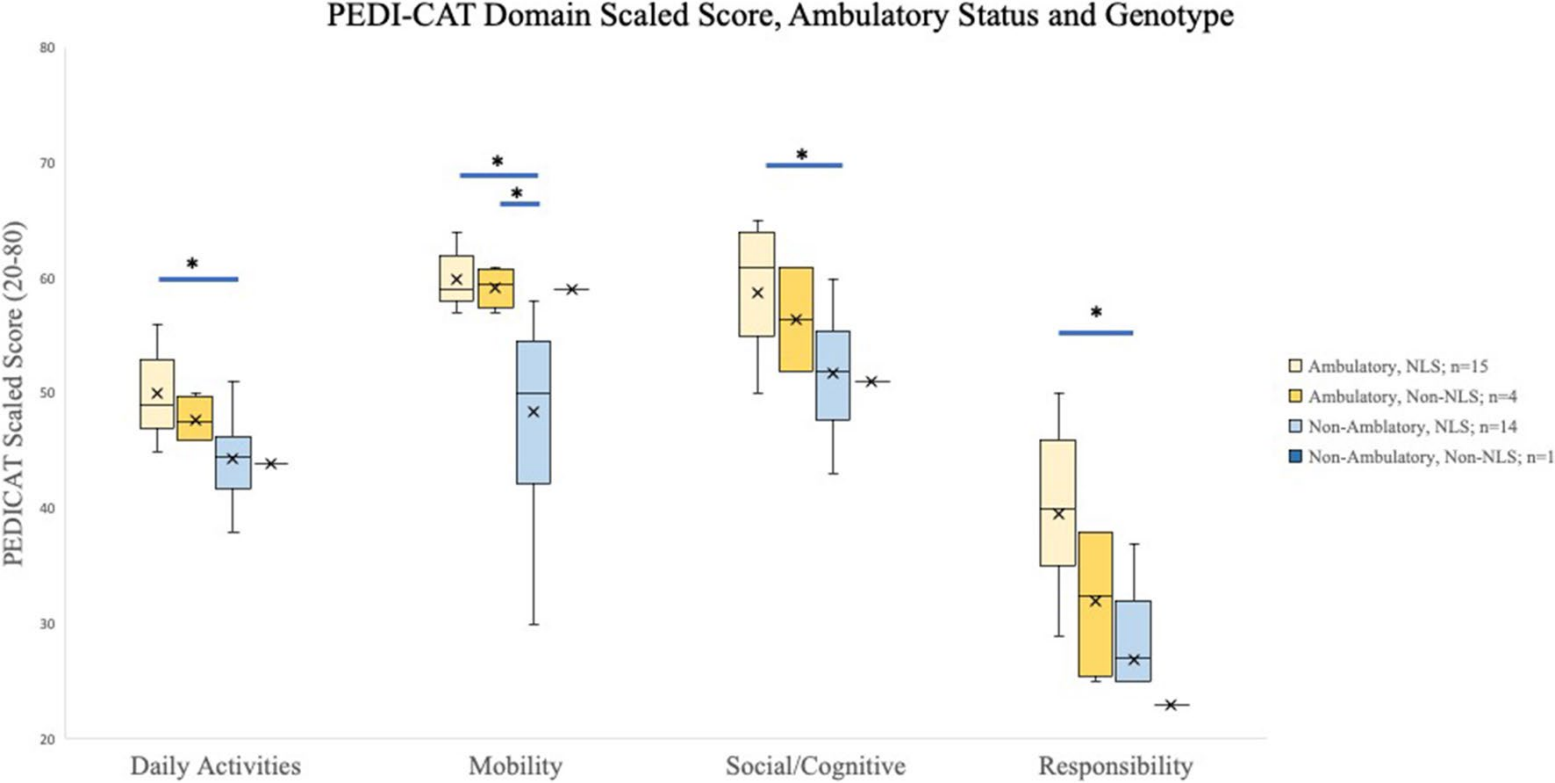
PEDI-CAT Domain Scaled Score, Ambulatory Status and Genotype. For each domain, error bars represent the 95% confidence interval, the bottom and top of the box are the 25th and 75th percentiles, the line inside the box is the 50th percentile (median). * denotes *p* < 0.05 using T-tests. A significant difference was seen between Ambulatory, NLS participants when compared to their non-ambulatory counterparts in all domains. A significant difference was seen between Ambulatory, Non-NLS participants compared to their non-ambulatory counterparts in only the Mobility Domain

When divided further, a significant difference was seen between ambulatory participants with variants inside the NLS region vs. non-ambulatory participants in all domains, however between for participants with variants outside of the NLS, a significant difference was only seen in the mobility domain when compared to their non-ambulatory counterparts (Fig. 5). The non-ambulatory/non-NLS participant could not be compared due to the small size of the group.

**Fig. 6:**
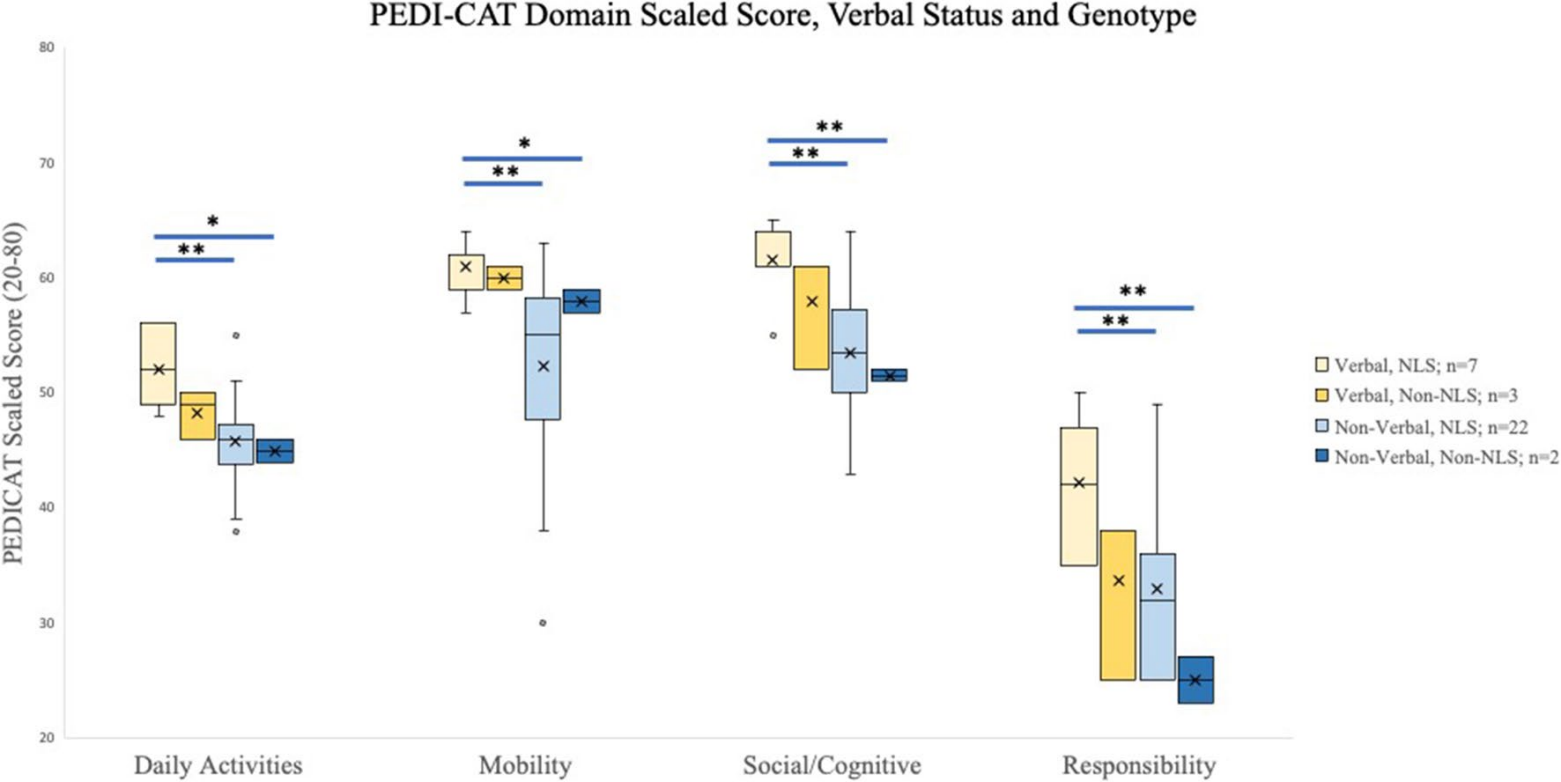
PEDI-CAT Domain Scaled Score, Verbal Status and Genotype. For each domain, error bars represent the 95% confidence interval, the bottom and top of the box are the 25th and 75^th^ percentiles, the line inside the box is the 50th percentile (median), and any outliers are shown as open circles. * denotes *p* < 0.05 and ** denotes *p* < 0.01 using T-tests. There was a significant difference seen between Verbal participants with variants in the NLS when compared to their to their non-verbal counterparts across all domains

Similarly, when verbal ability was separated by genotype, a significant difference was seen between verbal patients with variants inside the NLS compared to all non-verbal patients in all four PEDI-CAT domains. However, for verbal patients with variant outside of the NLS, no such difference was seen compared to non-verbal participants in any domain (Fig. 6).

### Longitudinal Analysis of PEDI-CAT Data

**Fig. 7:**
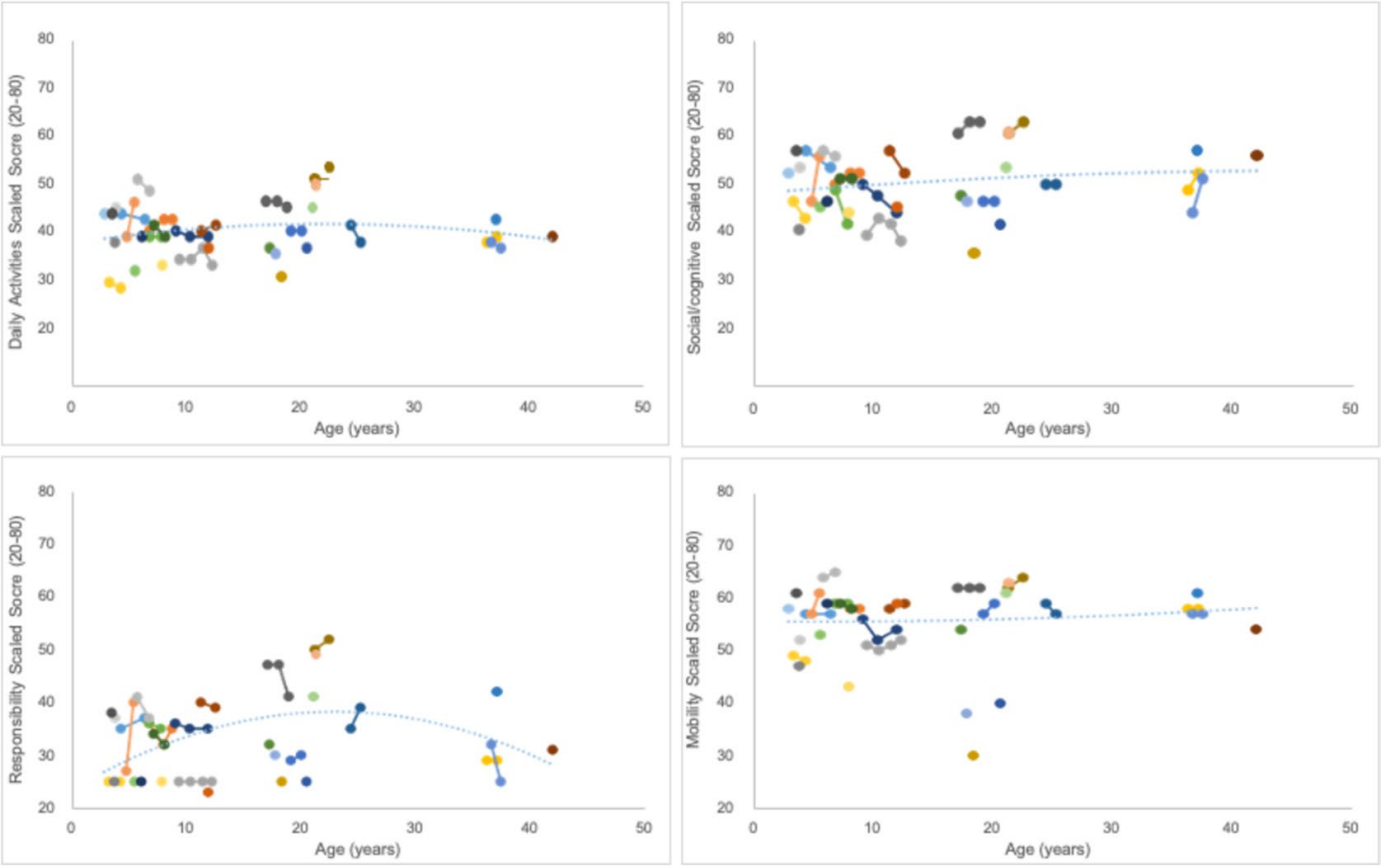
Longitudinal Trajectory of PEDI-CAT Scaled Scores by Domain. Individual participant data is linked represented by connected points. A trendline has been added across the cross-sectional sample suggesting stability across ages in Daily Activities, Social/Cognitive and Mobility, but possible regression in the Responsibility Domain

**Fig. 8:**
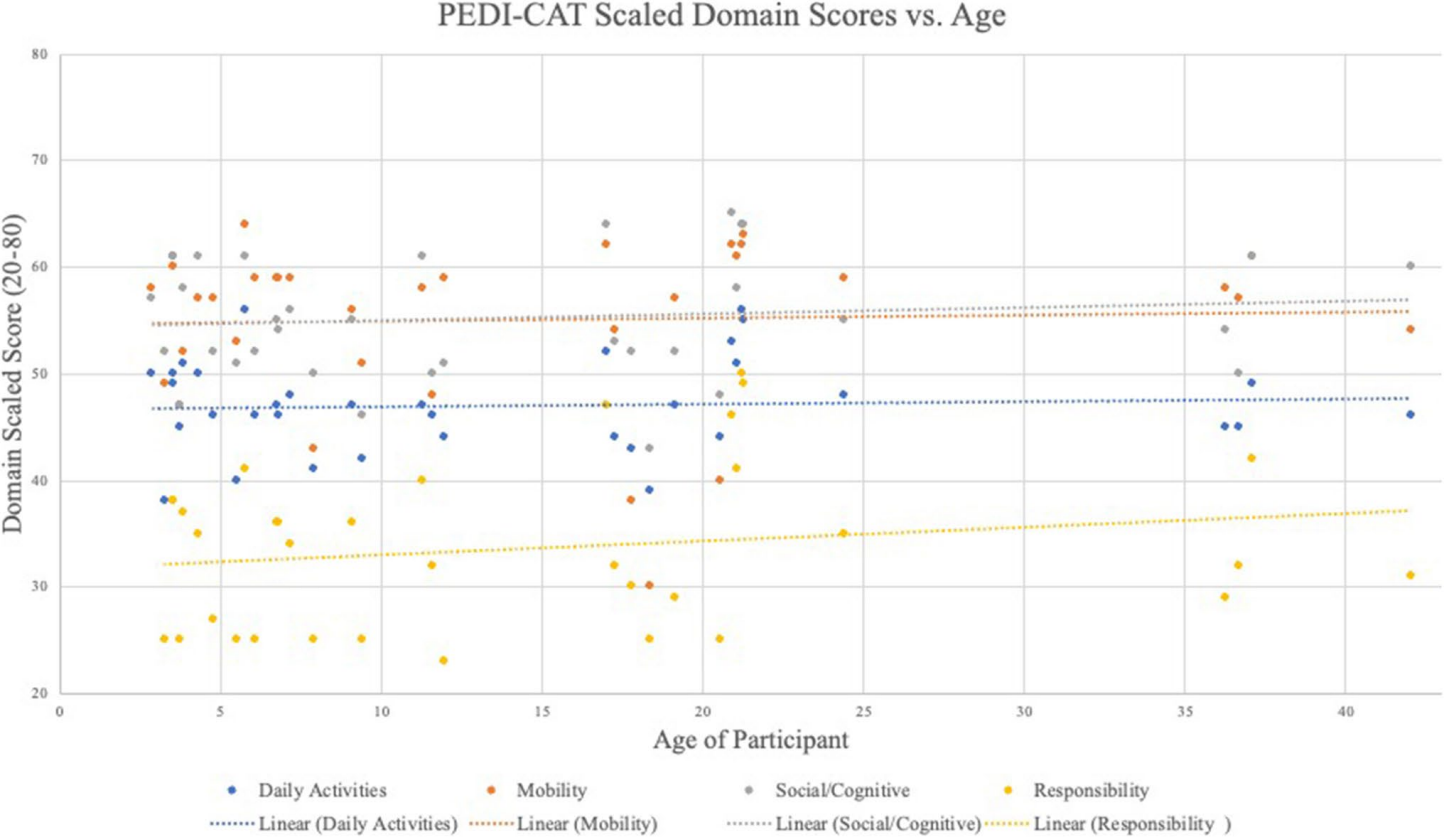
Individual Scaled Domain Score vs. Age of Participant at the Time of Survey. Each circle denotes an individual subject at a specific age. A trendline has been added to across the cross-sectional sample showing the lack of regression seen in this population as a function of their age

Of the 35 total individuals who completed the PEDI-CAT assessment, sixteen participants performed one year follow up evaluations, three completed an additional year follow up and two completed a fourth follow up evaluation. There was no significant difference seen between the PEDI-CAT scaled domain scores over time (Fig. 7). In addition, Looking at a cross-sectional sample of individual scaled scores demonstrate stability across ages, without a suggestion of regression using the PEDI-CAT measure (Fig. 8).

## Discussion

NDDs are a heterogenous group of disorders that result in a spectrum of cognitive, social, and motor abilities and ultimately may result in a high burden on individuals and caregivers. Identifying genetic variants that can cause these disorders can be important both for their care and for prognosis, helping caregivers understand those they care for and giving them information to set expectations and goals. It is therefore essential to have validated, reliable measures that can distinguish the clinical spectrum and assess the impact of therapeutic intervention. We have previously shown that parent-report measures are a valid-proxy for clinician-administered standardized motor evaluations in *HNRNPH2*-related NDD (Salazar et al., 2021) and we recognize that parent-report measures are integral to providing clinically meaningful feedback for rare NDDs. However, finding the appropriate measure for adaptive and cognitive skillsets may prove difficult as individual developmental disorders may have a broad phenotypic presentation even those with variants within the same gene.

The aim of this study was to expand the clinical phenotype of *HNRNPH2-* related NDD using parent-report measures. Two parent-report measures were used to capture the phenotypic spectrum of adaptive functioning in these individuals with *HNRNPH2-* related NDD. We first looked at the VABS-III and showed low overall scores across domains (Bain et al, 2021a, b). Then, given the initial descriptions of this disorder focused on the significant motor impairments (Bain et al., 2016, 2021a, b), we paid particular attention to deeply understand the motor deficits in this disorder and added the PEDI-CAT which is validated in an expanded age group as compared to the VABS-III. This study showed a strong correlation between an individual’s scores on the PEDI-CAT and VABS-III, notably seeing the composite VABS-III (ABC) score does seem to significantly correlate to the individual domains of the PEDI-CAT (mobility and social/cognitive). Additional studies may be important in future studies to focus on using the VABS-III parent-report measure.

Overall, individuals scored far below their age matched peers in all functional domains of the PEDI-CAT and VABS-III with norm-based scores (percentile, age-based scores), supporting findings from similar work using the VABS-III adaptive functioning on a small cohort of 31 individuals (Bain et al., 2021a, b). This raises concern about a potential floor effect wherein scores in the measured population fall too far below normative values decreasing the ability to interpret the results. This has been observed in other studies while using the PEDI-CAT tool (Dumas et al., 2021), so scaled scores can provide information about participant performance independent of the normative values to better understand the trends in the group and for an individual. By using the scaled scores, there is a wider distribution of individual scores, albeit lower than age-based norms seen in the scaled score. Additionally, individuals scored significantly lower on the Responsibility domain than the other three domains, Daily Activities, Mobility, and Social/Cognitive. This fourth domain (Responsibility) looks to the extent that an individual can take responsibility for tasks that are required for living independently and contains elements that draw from the other three domains. As such, this domain may not be particularly helpful to measure in this group of individuals due to the apparent floor effect of this domain in particular. Instead, one could look at individual tasks within the Responsibility Domain that may be more meaningful to this group or consider a disorder-specific survey that tailored questions to more appropriate goals for this group.

In trying to understand factors that cause significant differences in PEDI-CAT performance, scores were analyzed by age, verbal skills and mobility, and genotype. Not surprising, there were significant differences seen between verbal and non-verbal as well as ambulatory and non-ambulatory individuals. Importantly, the clinical picture, such as being ambulatory or verbal, seemed to align more with adaptive functioning than by looking at the genotype. Like other NDD studies, we suggest that the phenotypes of being verbal and/or ambulatory tend to have stronger overall adaptive function.

If we compared all individuals based upon their genetic variant location (within or outside the NLS), there was only a significant difference seen in the Motor Domain of the PEDI-CAT, however there was no significant difference in the Daily Activities, Social/Cognitive or Responsibility Domains. This may suggest the motor domain, representing impairments in motor functioning, may be particularly meaningful to differentiate between genotypes. In addition, if we looked at just the ambulatory individuals (or non-ambulatory), there is only a trend without statistical significance, between individuals with genotypes within versus outside the NLS (Fig. 5). This same insignificant trend is similar when looking at verbal individuals, comparing those with genotypes within or outside the NLS (Fig. 6). Though the sample sizes of each subgroup were very small, this highlights a potential difference in clinical phenotype that could correlate to genotype. In addition, all but one individual carried pathogenic missense variants, whereas one individual had a frameshift variant, presumably a loss of function variant. It will be interesting to expand on the clinical phenotype of individuals with missense versus loss of function variants if other genetic variants are further identified with a similar clinical phenotype. Further studies with larger cohorts are needed to better understand the relationship between functionality and specific genotypes, in addition to sex differences which were not studied here given the small cohort. Additionally, there is no additional medical co-morbidities or physical exam findings that correlated with the differences in adaptive skills. For example, the presence of seizures (or epilepsy) did not statistically differ between groups. Importantly, it will be worthwhile to investigate alternate methods of assessing adaptive function in those who are non-verbal and non-ambulatory to further identify meaningful clinical changes over time or between groups. More comprehensive language testing certainly would be important to further understand the communication complexities of this disorder as well with regards to the holistic clinical picture.

Lastly, one of the most important aspects of any longitudinal natural history project is understanding the baseline functional abilities of affected individuals as they grow. A cross-sectional age distribution does not show decline with age in three of the four domains (Fig. 7). As noted earlier, there may have been a basement effect using the Responsibility domain, which is also dependent upon the other 3 Domains. For this cross-sectional analysis, there appears to b a regression trend in this small group of older individuals. This may be due to the effect of having increased demands as an individual ages and will certainly need to be further studied. Lastly, in a small longitudinal sample, although limited in scale, shows a slight increase in participants scaled score without evidence of regression at three years (Fig. 8). Further studies with more longitudinal data, and a larger cohort may provide guidance about whether this is a statistically meaningful trend.

### Limitations and Future Directions

This study was limited by its small sample size, most notably seen in those who completed a follow up evaluation as well as the potential floor effects seen in the PEDI-CAT and VABS-III assessments due to the challenges of those with *HNRNPH2* related disorder.

As noted, assessing the raw scores, and looking at growth values, may provide more insight to clinically significant changes within individuals. Importantly, both the PEDI-CAT and VABS-III are parent/caregiver report measures, and while we have previously shown validity with the PEDI-CAT mobility domain by clinician-observation (Salazar et al., 2021), caregiver-reported surveys are likely without any specific input from the subjects who are nonverbal. Is remains essential to uphold the individual affected by a disorder as key stakeholder in natural history studies, despite the challenges to collect this information. Further evaluations of communication and language will be imperative to better understand the landscape of this skill in the *HNRNPH2*-related NDD group. As noted in the Methods, we did not differentiate between non-verbal and minimally verbal as the groups were already small and this study did not include formal cognitive and language testing. It will be critically important to better characterize this aspect of this NDD, as communication (in this study being noted by verbal status) appears to be correlating with overall adaptive functioning.

The future aim of this project is to continue to expand on our understanding of the clinical spectrum of this cohort with known pathogenic variants in *HNRNPH2*. As noted earlier, longitudinal data, including the use of raw data points using both the PEDI-CAT and the VABS-III may provide more tangible improvements in skills or losses in certain skills across time, as compared to normative data points which could point to losses or regression in comparison with age-equivalent peers.

Providing support to impacted families and caregivers is incredibly valuable and by expanding our knowledge about this cohort there can be a better understanding of the course of this disorder in all its variations. Understanding the limitations of using the PEDI-CAT and the VABS-III assessments in this population leads us to believe that it may be worthwhile to explore alternate measures of adaptive function. An alternate way to assess functional independence may be helpful for individuals and their families to measure clinically meaningful functional improvement seen after starting novel therapies. Additionally, while this limited survey showed no regression in this population, a longer time course of study will be beneficial to understand how the functional status of these individuals changes over time. This study illustrates the benefits of describing the adaptive functional capabilities in this population and the utility of using the PEDI-CAT and VABS-III tools for this purpose. In looking at the performance of this population on these assessments, this study has identified ways to continuously refine our measures to characterize this ultra-rare NDD.

## Data Availability

The datasets used and/or analyzed during the current study are available from the corresponding author on reasonable request.

## References

[CR1] Bain, J. M., Ardalan, A., & Goldman, S. (2021a). Deliberate paradigm shift in research in rare neurodevelopmental disorders. *Orphanet Journal of Rare Diseases,**16*(1), 263. 10.1186/s13023-021-01885-3 34107995 10.1186/s13023-021-01885-3 PMC8188744

[CR2] Bain, J. M., Cho, M. T., Telegrafi, A., Wilson, A., Brooks, S., Botti, C., Gowans, G., Autullo, L. A., Krishnamurthy, V., Willing, M. C., Toler, T. L., Ben-Zev, B., Elpeleg, O., Shen, Y., Retterer, K., Monaghan, K. G., & Chung, W. K. (2016). Variants in HNRNPH2 on the X Chromosome are associated with a neurodevelopmental disorder in females. *American Journal of Human Genetics,**99*(3), 728–734. 10.1016/j.ajhg.2016.06.028 27545675 10.1016/j.ajhg.2016.06.028 PMC5011042

[CR3] Bain, J. M., Snyder, L. G., Helbig, K. L., Cooper, D. D., Chung, W. K., & Goodspeed, K. (2022). Consistency of parent-report SLC6A1 data in simons searchlight with provider-based publications. *Journal of Neurodevelopmental Disorders,**14*(1), 40. 10.1186/s11689-022-09449-7 35761184 10.1186/s11689-022-09449-7 PMC9238190

[CR4] Bain, J. M., Thornburg, O., Pan, C., Rome-Martin, D., Boyle, L., Fan, X., Devinsky, O., Frye, R., Hamp, S., Keator, C. G., LaMarca, N. M., Maddocks, A. B. R., Madruga-Garrido, M., Niederhoffer, K. Y., Novara, F., Peron, A., Poole-Di Salvo, E., Salazar, R., Skinner, S. A., ... Chung, W. K. (2021b). Detailed clinical and psychological phenotype of the X-linked HNRNPH2-related neurodevelopmental disorder. *Neurology Genetics*, *7*(1), e551. 10.1212/nxg.0000000000000551 10.1212/NXG.0000000000000551 PMC7954461 33728377

[CR5] Berg, A. T., Palac, H., Wilkening, G., Zelko, F., & Schust Meyer, L. (2021). SCN2A-developmental and epileptic encephalopathies: challenges to trial-readiness for non-seizure outcomes. *Epilepsia,**62*(1), 258–268. 10.1111/epi.16750 33236786 10.1111/epi.16750

[CR6] Burgess, A., Boyd, R. N., Chatfield, M. D., Ziviani, J., & Sakzewski, L. (2020). Self-care performance in children with cerebral palsy: A longitudinal study. *Developmental Medicine & Child Neurology,**62*(9), 1061–1067. 10.1111/dmcn.14561 32430913 10.1111/dmcn.14561

[CR7] Burgess, A., Reedman, S., Chatfield, M. D., Ware, R. S., Sakzewski, L., & Boyd, R. N. (2022a). Development of gross motor capacity and mobility performance in children with cerebral palsy: A longitudinal study. *Developmental Medicine & Child Neurology,**64*(5), 578–585. 10.1111/dmcn.15112 34800033 10.1111/dmcn.15112

[CR8] Burgess, A., Sakzewski, L., Whittingham, K., Wotherspoon, J., Chatfield, M. D., Ware, R. S., & Boyd, R. N. (2022b). Development of social functioning in children with cerebral palsy: A longitudinal study. *Developmental Medicine & Child Neurology*, *n/a*(n/a). 10.1111/dmcn.15439 10.1111/dmcn.15439 PMC10953437 36282970

[CR9] Coster, W. J., Kramer, J. M., Tian, F., Dooley, M., Liljenquist, K., Kao, Y. C., & Ni, P. (2016). Evaluating the appropriateness of a new computer-administered measure of adaptive function for children and youth with autism spectrum disorders. *Autism,**20*(1), 14–25. 10.1177/1362361314564473 25630376 10.1177/1362361314564473 PMC4661128

[CR10] Dumas, H. M., Fragala-Pinkham, M. A., Haley, S. M., Ni, P., Coster, W., Kramer, J. M., Kao, Y.-C., Moed, R., & Ludlow, L. H. (2012). Computer adaptive test performance in children with and without disabilities: prospective field study of the PEDI-CAT. *Disability and Rehabilitation,**34*(5), 393–401. 10.3109/09638288.2011.607217 21988750 10.3109/09638288.2011.607217 PMC3668545

[CR11] Dumas, H. M., Fragala-Pinkham, M. A., Rosen, E. L., & Ni, P. (2021). A content validity evaluation of the PEDI-CAT Speedy Mobility domain. *Physiotherapy Theory and Practice,**37*(4), 517–526. 10.1080/09593985.2019.1633716 31232643 10.1080/09593985.2019.1633716

[CR12] Dumas, H. M., Fragala-Pinkham, M. A., Rosen, E. L., & O’Brien, J. E. (2017). Construct validity of the pediatric evaluation of disability inventory computer adaptive test (PEDI-CAT) in children with medical complexity. *Disability and Rehabilitation,**39*(23), 2446–2451. 10.1080/09638288.2016.1226406 27642790 10.1080/09638288.2016.1226406

[CR13] Duong, T. T. H., Goldman, S., Zhang, H., Salazar, R., Beenders, S., Cornett, K. M., Bain, J. M., Montes, J., & Zanotto, D. (2020). Validation of insole-based gait analysis system in young children with a neurodevelopmental disorder and autism traits. In: 2020 8th IEEE RAS/EMBS International Conference for Biomedical Robotics and Biomechatronics (BioRob)

[CR14] Farmer, C. A., Kaat, A. J., Thurm, A., Anselm, I., Akshoomoff, N., Bennett, A., Berry, L., Bruchey, A., Barshop, B. A., Berry-Kravis, E., Bianconi, S., Cecil, K. M., Davis, R. J., Ficicioglu, C., Porter, F. D., Wainer, A., Goin-Kochel, R. P., Leonczyk, C., Guthrie, W., Miller, J. S. (2020). Person ability scores as an alternative to norm-referenced scores as outcome measures in studies of neurodevelopmental disorders. *American Journal on Intellectual and Developmental Disabilities*, *125*(6), 475–480. 10.1352/1944-7558-125.6.475 10.1352/1944-7558-125.6.475 PMC11485197 33211814

[CR15] Garrido, D., Petrova, D., Watson, L. R., Garcia-Retamero, R., & Carballo, G. (2017). Language and motor skills in siblings of children with autism spectrum disorder: A meta-analytic review. *Autism Research,**10*(11), 1737–1750. 10.1002/aur.1829 28685955 10.1002/aur.1829

[CR16] Gillentine, M. A., Wang, T., Hoekzema, K., Rosenfeld, J., Liu, P., Guo, H., Kim, C. N., De Vries, B. B. A., Vissers, L. E. L. M., Nordenskjold, M., Kvarnung, M., Lindstrand, A., Nordgren, A., Gecz, J., Iascone, M., Cereda, A., Scatigno, A., Maitz, S., Zanni, G., ... Consortium, S. (2021). Rare deleterious mutations of HNRNP genes result in shared neurodevelopmental disorders. *Genome Medicine*, *13*(1), 63. 10.1186/s13073-021-00870-6 10.1186/s13073-021-00870-6 PMC8056596 33874999

[CR17] Hamburg, S., Lowe, B., Startin, C. M., Padilla, C., Coppus, A., Silverman, W., Fortea, J., Zaman, S., Head, E., Handen, B. L., Lott, I., Song, W., & Strydom, A. (2019). Assessing general cognitive and adaptive abilities in adults with Down syndrome: A systematic review. *Journal of Neurodevelopmental Disorders,**11*(1), 20. 10.1186/s11689-019-9279-8 31470792 10.1186/s11689-019-9279-8 PMC6716931

[CR18] Harmsen, S., Buchert, R., Mayatepek, E., Haack, T. B., & Distelmaier, F. (2019). Bain type of X-linked syndromic mental retardation in boys. *Clinical Genetics,**95*(6), 734–735. 10.1111/cge.13524 30887513 10.1111/cge.13524

[CR19] Hu, W. F., Chahrour, M. H., & Walsh, C. A. (2014). The diverse genetic landscape of neurodevelopmental disorders. *Annual Review of Genomics and Human Genetics,**15*, 195–213. 10.1146/annurev-genom-090413-025600 25184530 10.1146/annurev-genom-090413-025600 PMC10591257

[CR20] Jepsen, W. M., Ramsey, K., Szelinger, S., Llaci, L., Balak, C., Belnap, N., Bilagody, C., De Both, M., Gupta, R., Naymik, M., Pandey, R., Piras, I. S., Sanchez-Castillo, M., Rangasamy, S., Narayanan, V., & Huentelman, M. J. (2019). Two additional males with X-linked, syndromic mental retardation carry de novo mutations in HNRNPH2. *Clinical Genetics,**96*(2), 183–185. 10.1111/cge.13580 31236915 10.1111/cge.13580 PMC6852257

[CR21] Kalb, L., Jacobson, L., Zisman, C., Mahone, E., Landa, R., Azad, G., Menon, D., Singh, V., Zabel, A., & Pritchard, A. (2019). Interest in research participation among caregivers of children with neurodevelopmental disorders. *Journal of Autism and Developmental Disorders,**49*(9), 3786–3797. 10.1007/s10803-019-04088-9 31172337 10.1007/s10803-019-04088-9 PMC6669084

[CR22] Koegel, L. K., Bryan, K. M., Su, P. L., Vaidya, M., & Camarata, S. (2020). Definitions of nonverbal and minimally verbal in research for autism: a systematic review of the literature. *Journal of Autism and Developmental Disorders,**50*(8), 2957–2972. 10.1007/s10803-020-04402-w 32056115 10.1007/s10803-020-04402-w PMC7377965

[CR23] Kreienkamp, H.-J., Wagner, M., Weigand, H., McConkie-Rossell, A., McDonald, M., Keren, B., Mignot, C., Gauthier, J., Soucy, J.-F., Michaud, J. L., Dumas, M., Smith, R., Löbel, U., Hempel, M., Kubisch, C., Denecke, J., Campeau, P. M., Bain, J. M., & Lessel, D. (2022). Variant-specific effects define the phenotypic spectrum of HNRNPH2-associated neurodevelopmental disorders in males. *Human Genetics,**141*(2), 257–272. 10.1007/s00439-021-02412-x 34907471 10.1007/s00439-021-02412-x PMC8807443

[CR24] Krishnan, M. L., Berry-Kravis, E., Capal, J. K., Carpenter, R., Gringras, P., Hipp, J. F., Miller, M. T., Mingorance, A., Philpot, B. D., Pletcher, M. T., Rotenberg, A., Tjeertes, J., Wang, P. P., Willgoss, T., de Wit, M. C., & Jeste, S. S. (2021). Clinical trial strategies for rare neurodevelopmental disorders: Challenges and opportunities. *Nature Reviews. Drug Discovery,**20*(9), 653–654. 10.1038/d41573-021-00085-9 34002058 10.1038/d41573-021-00085-9

[CR25] Levine, J., Hakim, F., Kooy, R. F., & Gozes, I. (2022). Vineland adaptive behavior scale in a cohort of four ADNP syndrome patients implicates age-dependent developmental delays with increased impact of activities of daily living. *Journal of Molecular Neuroscience,**72*(8), 1531–1546. 10.1007/s12031-022-02048-0 35920977 10.1007/s12031-022-02048-0

[CR26] Manickam, K., McClain, M. R., Demmer, L. A., Biswas, S., Kearney, H. M., Malinowski, J., Massingham, L. J., Miller, D., Yu, T. W., Hisama, F. M., & Directors, A. B. O. (2021). Exome and genome sequencing for pediatric patients with congenital anomalies or intellectual disability: an evidence-based clinical guideline of the American College of Medical Genetics and Genomics (ACMG). *Genetics in Medicine,**23*(11), 2029–2037. 10.1038/s41436-021-01242-6 34211152 10.1038/s41436-021-01242-6

[CR27] Mouga, S., Almeida, J., Café, C., Duque, F., & Oliveira, G. (2015). Adaptive profiles in autism and other neurodevelopmental disorders. *Journal of Autism and Developmental Disorders,**45*(4), 1001–1012. 10.1007/s10803-014-2256-x 25241010 10.1007/s10803-014-2256-x

[CR28] Posar, A., & Visconti, P. (2021). Update about “minimally verbal” children with autism spectrum disorder. *Revista Paulista de Pediatria,**40*, e2020158. 10.1590/1984-0462/2022/40/2020158 34495269 10.1590/1984-0462/2022/40/2020158 PMC8432069

[CR29] Rare Diseases: Natural History Studies for Drug Development. (2019). Website with FDA draft guidance: https://www.fda.gov/regulatory-information/search-fda-guidance-documents/rare-diseases-natural-history-studies-drug-development

[CR30] Salazar, R., Beenders, S., LaMarca, N. M., Thornburg, O., Rubin-Thompson, L., Snow, A., Goldman, S., Chung, W. K., & Bain, J. M. (2021). Cross-sectional, quantitative analysis of motor function in females with HNRNPH2-related disorder. *Research in Developmental Disabilities,**119*, 104110. 10.1016/j.ridd.2021.104110 34794115 10.1016/j.ridd.2021.104110

[CR31] Shore, B. J., Allar, B. G., Miller, P. E., Matheney, T. H., Snyder, B. D., & Fragala-Pinkham, M. (2019). Measuring the reliability and construct validity of the pediatric evaluation of disability inventory-computer adaptive test (PEDI-CAT) in children with cerebral palsy. *Archives of Physical Medicine and Rehabilitation,**100*(1), 45–51. 10.1016/j.apmr.2018.07.427 30130519 10.1016/j.apmr.2018.07.427

[CR32] Tasse, M. J., Schalock, R. L., Balboni, G., Bersani, H., Jr., Borthwick-Duffy, S. A., Spreat, S., Thissen, D., Widaman, K. F., & Zhang, D. (2012). The construct of adaptive behavior: Its conceptualization, measurement, and use in the field of intellectual disability. *American Journal on Intellectual and Developmental Disabilities,**117*(4), 291–303. 10.1352/1944-7558-117.4.291 22809075 10.1352/1944-7558-117.4.291

[CR33] Vorstman, J. A. S., Parr, J. R., Moreno-De-Luca, D., Anney, R. J. L., Nurnberger, J. I., Jr., & Hallmayer, J. F. (2017). Autism genetics: Opportunities and challenges for clinical translation. *Nature Reviews Genetics,**18*(6), 362–376. 10.1038/nrg.2017.4 28260791 10.1038/nrg.2017.4

